# Study protocol for a randomized double-blinded placebo-controlled trial on mepolizumab for patients with chronic rhinosinusitis with nasal polyps, NSAID exacerbated respiratory disease and asthma

**DOI:** 10.3389/falgy.2025.1568081

**Published:** 2025-03-21

**Authors:** Annina Lyly, Johanna Sahlman, Karoliina Pajala, Maija Salminen, Saara Sillanpää, Jura Numminen, Tanzeela Hanif, Anu Laulajainen-Hongisto, Mika Mäkelä, Paula Kauppi, Iiris Kangasniemi, Markus Lilja, Sari Hammaren-Malmi, Paula Virkkula, Sanna Toppila-Salmi

**Affiliations:** ^1^ Department of Allergology, Inflammation Center, Helsinki University Hospital and University of Helsinki, Helsinki, Finland; ^2^ Department of Otorhinolaryngology–Head and Neck Surgery, Helsinki University Hospital and University of Helsinki, Helsinki, Finland; ^3^ Department of Otorhinolaryngology, University of Eastern Finland, Joensuu, Kuopio, Finland; ^4^ Department of Otorhinolaryngology, Wellbeing Services County of Pohjois-Savo, Kuopio, Finland; ^5^ Department of Otorhinolaryngology, Head and Neck Surgery, Tampere University Hospital, Tampere, Finland; ^6^ Wellbeing Services County of Pirkanmaa, Faculty of Medicine and Health Technology, Tampere University, Tampere, Finland; ^7^ Heart and Lung Center, Department of Pulmonary Diseases and Allergology, Helsinki University Hospital and University of Helsinki, Helsinki, Finland

**Keywords:** asthma, NERD, CRSwNP, mepolizumab, biologics

## Abstract

**Background:**

Chronic rhinosinusitis with nasal polyps (CRSwNP) is an inflammatory disease of the nose and paranasal sinuses that significantly impactshealth-related quality of life. Nonsteroidal anti-inflammatory drug (NSAID) -exacerbated respiratory disease (N-ERD) affects approximately one fifth of CRSwNP patients. N-ERD and asthma increase the risk of uncontrolled CRSwNP as measured by frequent sinus surgeries and rescue treatment. Compared to non-N-ERD patients, those with N-ERD also have higher risk of asthma exacerbations, severe allergic reactions, and anosmia. Mepolizumab is a humanized monoclonal anti-IL-5 antibody shown to be effective in treating severe eosinophilic asthma and CRSwNP. While evidence suggests that mepolizumab alleviates respiratory symptoms in N-ERD patients, placebo-controlled studies remain limited.

**Methods:**

The aim of this prospective randomized, placebo-controlled, multicenter study is to investigate whether mepolizumab reduces polyp size, symptom scores, and exacerbations more than placebo during the 16-week treatment period in patients with uncontrolled CRSwNP, N-ERD and asthma. Additionally, we will examine the effect of mepolizumab on drug dosage and lung and nasal function and evaluate predictive biomarkers.

We will recruit 120 patients with N-ERD, nasal polyposis and asthma in three centers in Finland. Patients will be randomized into two 16-week treatment groups in 1:1 ratio (placebo or mepolizumab 100 mg every 4 weeks). The study lasts for 6 months, including recruitment visit 2–4 weeks before randomization. Participants will attend 6 visits, during four of which they will receive a subcutaneous injection of the study product. At each visit, patient-reported outcome tests, clinical examination, airway function tests, and nasal, blood, urine, and stool samples will be conducted.

**Discussion:**

The efficacy of the 16-week anti-IL-5-treatment in this severe patient group will be analyzed, as well as possible predictive biomarkers.

**Clinical Trial registration:**

ClinicalTrials.gov ID NCT04823585. Registered on 28.3.2021.

## Background

Chronic rhinosinusitis (CRS) is a symptomatic inflammatory disease of the nasal and paranasal mucosa lasting more than 12 weeks (1). It is classified into two phenotypes: polypotic (CRSwNP) and non-polypotic (CRSsNP). Asthma is a prolonged bronchial inflammatory disease with an increased and variable tendency for bronchial contraction (2). CRS and asthma often coexist and affect each other's disease control (3).

Approximately one-fifth of people with CRS and/or asthma have a severe form of the disease in which the standard treatment is not enough to achieve disease control. The costs associated with severe CRS and asthma account for the majority of the total costs for the entire disease group (1, 4, 5). Severe disease is typically characterized by eosinophilic airway inflammation and recurrent purulent exacerbations. Patients often present with concomitant CRSwNP, asthma, and non-steroidal anti-inflammatory drug (NSAID)-exacerbated respiratory disease (N-ERD) with exacerbation triggered by acetylsalicylic acid (ASA) or another NSAID (6). These patients have an increased frequency of surgeries, a higher risk of complications, and a greater likelihood of side effects from other treatments, such as recurrent systemic corticosteroids or experimental ASA desensitization therapy are increased in these patients (7).

The mucous membranes of the airways regulate airflow, transport inhaled air, and serve as sensory and defensive barrier against airborne particles. Inflammatory responses against pathogens are categorized by the type of the following immune reactions. Type 2 inflammatory mediators, including IL-4, IL-5, and IL-13, normally protect against helminth infections, but are upregulated in many chronic airway inflammatory diseases (8). Patients with N-ERD experience exaggerated type 2 airway inflammation, leading to symptoms such as excessive mucus production, cough, shortness of breath, congestion, impaired sense of smell, fatigue, and recurrent exacerbations (6). The pathomechanisms of N-ERD remain poorly understood. An imbalance in mucosal microbiota, along with dysregulation of the immune system and genetic factors may contribute to disease development. A study in the Icelandic/English population showed that *ALOX15* gene variant is associated with a lower prevalence of CRSwNP (9), though other genetic factors remain largely unknown. Further research on these factors could help identify high-risk patients and enable more targeted treatment approaches in the future.

Targeted biologic therapy represents a significant advancement in improving outcomes for type 2 inflammatory diseases, including N-ERD (10). Biologics are typically considered after the relapse of advanced treatments such as endoscopic sinus surgery, oral corticosteroid courses or acetylsalicylic acid (ASA) therapy after desensitization (ATAD). Biologic therapy has been recommended to patients with contraindications of ASA therapy such as a history of gastritis, bleeding risk, or severe uncontrolled asthma (11). There remains an unmet need for further research into personalized treatment strategies and the identification of the most effective biologic therapy for N-ERD.

Mepolizumab is a humanized monoclonal IgG antibody that targets interleukin 5 (IL-5) and blocks its binding to its receptor. IL-5, produced especially by Th2 lymphocytes, is a key cytokine in eosinophil-mediated inflammation. IL-5 regulates eosinophil differentiation, growth, activation, survival, and migration to airways. Inhibiting IL-5 reduces blood eosinophil levels and eosinophilic inflammation (12).

Mepolizumab has demonstrated efficacy in the treatment of severe eosinophilic asthma (13) and CRSwNP (14). While several retrospective cohorts and reviews have examined the effects of biologics on N-ERD patients, to the authors' knowledge, no randomized prospective studies on mepolizumab for N-ERD patients have been conducted.

The aim of this prospective randomized, double-blinded, placebo-controlled, multicenter study is to evaluate the safety and effectiveness of mepolizumab in reducing polyp size, symptom scores and exacerbations in patients with uncontrolled CRSwNP, N-ERD and asthma. Additionally, the study will assess the impact of mepolizumab on drug dosage, lung and nasal function and search for predictive biomarkers. Although mepolizumab has been shown to be effective in N-ERD, we believe it is important to investigate its effectiveness and safety in the Finnish N-ERD population, which has previously shown poor tolerability of ASA therapy (15).

## Methods/design

The objective of the study is to investigate the effects of mepolizumab in the treatment of patients with uncontrolled CRSwNP, N-ERD and asthma

• Does mepolizumab reduce endoscopic nasal polyp score and SNOT-22 symptom score more than placebo?
• Does mepolizumab reduce exacerbation-rate, reduce need for dosing-up medication, reduce signs of Th2-inflammation (such as eosinophilia) in blood and nasal samples, as well as improve lung and nasal function more effectively than placebo?

The study is conducted as a randomized double-blinded prospective controlled multicenter trial. A total of 120 adult N-ERD patients will be recruited. Approximately 70–75 patients will be recruited at the Departments of Otorhinolaryngology and Allergy of Helsinki University Hospital, and the rest will be recruited at the Departments of Otorhinolaryngology, Allergy and Pulmonology of Tampere University Hospital (*N* = 35) and Kuopio University Hospital (*N* = 10–15). The study will be monitored by a professional monitor. Electronic case report forms (eCRF) and paper/electronic patient questionnaires provided by HUS will be used (eCRF and patient questionnaire, Granitics).

The diagnosis of CRSwNP is based on a positive history of CRSwNP, nasal endoscopy, and computed tomography scans (16). EPOS-criteria for CRS include inflammation of the nose and the paranasal sinuses characterized by two or more symptoms, one of which should be either nasal blockage/obstruction/congestion or nasal discharge (anterior/posterior nasal drip): ± facial pain/pressure, ± reduction or loss of smell.

Disease-specific quality of life is evaluated using the SNOT-22 questionnaire, a well-established tool for assessing the physical, functional, and emotional impact of CRS. It is a reliable and effective method for detecting changes resulting from interventions. The minimal clinically important difference (MCID) for SNOT-22 is 8, 9 points (17). The nasal polyp score (NPS) is assessed using the modified Davos Scale, which ranges from 0 to 4 per side (0–8 total) (18). The MCID for the NPS is 1 point (19).

The diagnosis of asthma is based on typical history, clinical features, and at least one of the following physiologic criteria: (i) a recurrent variation of 20% or greater in diurnal peak expiratory flow (PEF) recording (reference to diurnal mean); (ii) a recurrent increase of 15% or greater in PEF with β-agonist or (iii) an increase of 12% or greater in forced expiratory volume in 1 s (FEV1) with β-agonist; or (iv) a decrease of 15% or greater in PEF or FEV1 in exercise testing or moderate to severe hyperresponsiveness in a bronchial provocation test.

N-ERD is confirmed by typical history and by ASA challenge test unless there is previous report of NSAID-provoked anaphylaxis or acute airway attack leading to hospitalization, or a previous positive ASA challenge test at a hospital setting. For patients without a prior ASA challenge, the test is conducted in a hospital setting where they receive 50 mg of ASA. Patients are monitored by airway function tests, questionnaires, and clinical examination. The patients are followed for at least 4 h.

Patients who do not have N-ERD will not be included in the trial. All patients entering the trial must have undergone at least one prior endoscopic sinus surgery (ESS) and have failed to achieve disease control. Clinical baseline data, including smoking, allergy, asthma, medication, previous operations, co-morbidities, and symptom duration, will be collected

Inclusion criteria:

• ≥18 years of age
• exacerbation of respiratory symptoms by ASA or another NSAID. N-ERD will be verified by another visit if necessary.
• CRS with bilateral polyps. Endoscopic nasal polyp score of at least 5 out of 8, with a minimum score of 2 in each nasal cavity (e.g., not RIGHT 1 and LEFT 4 or vice versa).
• Lund Mackay score ≥12 (maximum 24) of sinus computed tomography (CT) or cone beam (CBCT) scans. New sinus CT/CBCT scans are needed if the previous scans have been performed over 36 months before recruitment visit or if there is a suspicion of complication of CRS (e.g., mucocele, invasive fungal rhinosinusitis). The data of previous sinus CT/CBCT scans will be used if the scans have been performed ≤36 months prior to recruitment visit.
• females of reproductive potential (FRPs) who are not pregnant or breast-feeding may be enrolled. FRPs need to perform pregnancy test prior to the CT/CBCT scans. If the subject is already on contraception prior to the study this should be continued.
• ≥1 previous ESS performed. The last operation must have been performed at least 6 months before the 1st visit.
• SNOT-22 ≥25
• at least one other symptom, such as partial or total loss of smell (hyposmia or anosmia), nasal obstruction, or anterior or posterior rhinorrhea
• history of at least one exacerbation during the past 2 years, e.g., at least one criterion must be fulfilled of the following list during the past 2 years: ≥1 oral corticosteroids; ≥3 antibiotic courses; ≥1 ESS; ≥  asthma hospitalization. In patients with contraindications of previously listed treatments or continuous oral steroids, additional criteria are not required.
• asthma diagnosis
• peripheral blood eosinophils (B-Eos) >300 cells/ul at visit 1 OR (B-Eos >150 cells/ul at visit 1 AND a history of B-Eos >300 cells/ul during the past 12 months). A history of nasal polyp tissue eosinophilia (NP-Eos) ≥30% during the past 12 months is a supportive criterion.
• regular use of intranasal corticosteroid started at least 2 months prior to study entry. Regular CRSwNP and asthma medication is used throughout the whole study.

Exclusion criteria:

• Age <18 years
• ESS <6 months before the 1st visit
• pregnancy breastfeeding
• complication of CRS, e.g., mucocele, invasive fungal rhinosinusitis
• acute rhinosinusitis/respiratory infection
• severe disease related to airways/immunology: cystic fibrosis, primary ciliary dyskinesia, sarcoidosis, immunosuppression, diagnosed specific antibody deficiency, common variable immunodeficiency, human immunodeficiency virus, fungal rhinosinusitis, Young syndrome, Kartagener syndrome
• other severe disease such as active cancer
• received biologic therapy/systemic immunosuppressant/ASA desensitization therapy/experimental monoclonal antibody treatment to treat inflammatory or autoimmune disease within 2 months of study entry or 5 half-lives, whichever is longer. The patient is allowed to use ASA dose <100 mg/day due to cardiovascular reasons after ASA desensitization.
• current immunotherapy
• communication problems (e.g., neurological/psychiatric disease, language skills)
• unlikely to comply
• ASA-challenge negative
• history of hypersensitivity to mepolizumab or excipients in the formulation

ASA challenge is conducted during the second visit if needed, according to modified international protocol (16, 17). FEV1 should be at least 1.5 L and >60% of predicted before challenge or desensitization. Patients will receive 25 mg of ASA at a hospital setting. If no remarkable symptoms are seen, patients will receive another 25 mg of ASA 1–2 h later.

ASA challenge positive result:

1. Naso-ocular alone: a 30% or >increase in at least one of the following VAS (0–10 cm) scores: nasal obstruction, nasal discharge, postnasal drip, eye itching. These may exist with or without objective signs of increased nasal turbinate swelling/discharge or eye redness in examination.
2. Naso-ocular (please see 1.) and a 15% or >decline in FEV1 or in PEF (Classic reaction)
3. Lower respiratory reaction only (FEV1 or PEF declines by >20%)
4. Laryngospasm with or without 1–3 (flat or notched inspiratory curve)
5. Systemic reaction: hives, flush, gastric pain, hypotension

Patient randomization occurs after the 1st (or 2nd for ASA challenged) visit. Patients are randomized into two treatment arms: (I) subcutaneous injections of placebo once per month for 16 weeks (II) subcutaneous injections of mepolizumab once per month for 16 weeks. Mepolizumab 100 mg/Placebo (NaCl solution) is injected subcutaneously once per month, for 16 weeks. GSK provides randomization codes to each center.

Power calculation: The MCID for SNOT-22 is 8, 9 points (17). Therefore, a mean difference was assumed to be at least 9 between the treatment arms. In a previous study the response within each subject group was normally distributed with a standard deviation of 17. If the true difference in means between the experimental and control groups is is 13, 57 subjects per group (experimental and control) are required to reject the null hypothesis that the population means are equal with probability (power) 0.8. The Type I error probability associated with this test is 0.05. The total number recruited is 120, accounting for an estimated 5% discontinuation rate.

### Study endpoints

#### Primary objectives

The primary aims of this study are to evaluate the relative change of SNOT-22 and endoscopic nasal polyp score (NPS) at 5 months vs. baseline in the two randomized treatment arms (Table 1).

**Table 1:** Study protocol.

Time	−8 weeks	−4 weeks	0 weeks	4 weeks	8 weeks	12 weeks	16 weeks	20 weeks
Visit	1	2	3	4	5	6	7	8
Name of the visit	Recruitment	ASA-challenge	1st injection	2nd injection	3rd injection	4th injection	Follow-up visit	End of trial
Informed consent	x	–	–	–	–	–	–	–
Review inclusion/exclusion criteria	x	x	x	–	–	–	–	–
Medical history comorbidities	x	–	–	–	–	–	–	–
Intranasal fluticasone propionate (FP) drops or corticosteroid spray twice daily	x	x	x	x	x	x	x	x
Randomization	–	–	x	–	–	–	–	
Visiting nurse	x	x	x	x	x	x	x	x
Review of concomitant Medication	x	x	x	x	x	x	x	x
Visits, hospitalisations, exacerbations, sick leave, productivity	x Previous year	x	x	x	x	x	x	x
ASA challenge test (to subjects without previous challenge-test-confirmed NERD dg)	–	x	–	–	–	–	–	
Treatment with IMP	–	–	x	x	x	x	–	
Check side-effects, adverse event enquiry	–	x	x	x	x	x	x	x
Visiting doctor (ENT, pulmonology consultation if needed)	x	x	x	x	x	x	x	x
Check CRS control	x	x	x	x	x	x	x	x
Pulmonologist Consultation	x	–	–	–	–	–	x	
Check asthma control	x	x	x	x	x	x	x	x
Questionnaire-packet: SNOT-22, 15D, mini-AQLQ, Asthma control test, iMCQ,iPCQ, WPAI, EQ-5D-5l	x	x	x	x	x	x	x	x
VAS symptoms	x	x	x	x	x	x	x	x
Baseline characters questionnaire	x	–	–	–	–	–	–	(x) if changed
Acoustic rhinometry (ARM)	–	(xx)	(x) ARM if not ASA-challenge	–	–	–	x	–
Nasal endoscopy	x	x	x	x	x	x	x	X
eNO	x	–	–	–	–	–	x	
Olfaction Sniffin’ sticks	x	–	x	x	x	x	x	x
Sinus CT/CBCT scans	(x) if needed (see inclusion criteria)	–	–	–	–	–	–	
Blood samples	x	xx	x	x	x	x	x	x
Urine samples	x	xx	x	x	x	x	x	x
Fecal samples	–	–	x	–	–	–	x	–
Nasal samples	x	xx	–	–	–	–	x	
Spirometry	x	–	–	–	–	–	x	
PEF 1 week	x	x	–	–	–	–	x	
Microspirometry	x	xx	x	x	x	x	x	x
PEF at visit	x	xx	x	x	x	x	x	x
PNIF at visit	x	x***x***	x	x	x	x	x	x
Consider additional therapy/referral to operation	x	x	x	x	x	x	x	x

#### Secondary objectives

To evaluate the relative change of the following parameters between the two treatment arms:

1. VAS score (smell loss, obstruction, postnasal drip, nasal discharge, facial pain/pressure)
2. exacerbation rate. Exacerbation means that patient has at least one of the following due to airway symptoms: oral corticosteroid course; antibiotic course; sinus lavation; hospitalization.
3. other medication usage such as dosing-up medication, and medication of exacerbations (antibiotics, bronchodilators, nasal decongestants, systemic corticosteroids)
4. number of patients who met criteria for requiring surgery for polyposis at each time point, or consideration/planning of an operation due to airway symptoms
5. signs of type 2 inflammation in blood and nasal samples (B-Eos, NP-Eos)
6. nasal and lung function test results [FEV1, peak expiratory flow rate, exhaled NO, acoustic rhinometry, peak nasal inspiratory flow (PNIF), Smell test by Sniffin' sticks 12]
7. general and disease-specific quality of life questionnaires: 15D, mini asthma quality of life questionnaire (mini-AQLQ), asthma control test (ACT), and EQ-5D-5l
8. cost-effectiveness and productivity: medical consumption questionnaire (iMCQ), productivity cost questionnaire (iPCQ), work productivity and activity impairment questionnaire (WPAI), sick leaves due to airway symptoms
9. safety (adverse events)

The randomization will be removed, and patients will be informed about their treatment arm after the trial has been terminated. The treatment of the patients continues as per local guidelines and practice after the trial.

### Reporting and monitoring of adherence to treatment

Adherence of the treatment of IMP is ensured by performing the subcutaneous injection by a well-trained study nurse at each visit. To monitor adherence of other CRSwNP and asthma treatments, patients' medication use, dosages, and compliance are assessed and recorded by both the doctor and the study nurse at each visit. Additionally, medication adherence is tracked through the national electronic prescription database, which allows us to monitor all prescribed medications purchased by the patients.

### Reporting and monitoring of adverse events and reactions

Adverse events during the trial are documented. Side effects of the investigational medicinal product are recorded throughout the follow-up. Any adverse events during the trial are recorded in accordance with the 2017 updated Guideline for Good Clinical Practice (GCP) (EMA/CPMP/ICH/135/95). Pharmaceutical manufacturer GlaxoSmithKline (GSK) will receive a safety report from the research unit regarding any incidents.

### Follow-up of the patient after the study

There is no follow-up after the study in the research protocol. However, the follow-up monitoring is performed according to normal clinical routine, as the recruited patients are followed by their tertiary clinics, in which the study centers are also located. Patients who discontinue participation are asked for permission to use the data collected.

### Data management

#### Statistics

Data analysis will be carried out on the relationship between mepolizumab and placebo treatment. Variables tested in the statistical models include age, gender, symptoms, exacerbations, infections, medications, endoscopic findings, sick leave, allergy, side-effects of treatment, hospitalizations, and other factors at different time-points. Student's *t*-test (or KW, MWU tests, in case of not normally distributed sample), as well as adjusted linear regression models are used for continuous measures. *post hoc* analyses with inclusion of subjects with higher baseline mean SNOT-22 score will be performed. A statistical analysis plan (such as correction for multiple testing) will be drawn up with advice from the statistical department at the University of Helsinki.

We will use several software: R 3.0.2, SPSS 22.0 Statistical Software Package (SPSS, Chicago, IL) and STATA (Release 13.1 software, StataCorp, Texas, USA). For comparisons, the results will be analyzed by Logistic regression, Student's *t*-, Chi-square, (nonparametric) Fisher's exact and Mann Whitney *U* test. *P*-values less than 0.05 are considered statistically significant. Results of logistic regression will be reported as odds ratios (OR) with 95% confidence intervals. All models will be adjusted by selected covariates (potential confounding factors).

### Sample handling

The samples will be stored at Helsinki Biobank or at the University of Helsinki. We aim at performing exome sequencing, mRNA sequencing and metatranscriptomics, immunohistochemistry, Western blot and ELISA, metabolomics to search for biological key factors relevant for aggravated CRS and asthma and N-ERD. For exome sequencing, DNA will be isolated from peripheral blood leukocytes, PCR enriched, and libraries will be processed and sequenced on an Illumina HiSeq. For microbiome, 16S-RNA sequencing and metatranscriptomics will be performed to several sample types (nasal, nasopharynx, fecal). Reads will be mapped to rRNA reference sequences and outputs will be summarized for each phylotype. For DNA profiling, raw bisulphite treatment will be performed on DNA. Oligonucleotide primers will be synthesized. Libraries will be processed and sequenced. SNPs and indels will be called and annotation is performed by ANNOVAR. For transcriptomics, total RNA from nasal cell brushings will be extracted, enriched RNA-seq library will be prepared and Nextera Primers are used. miRNA is PCR enriched, and small RNA libraries sequenced. Reads will be corrected and aligned to the reference human genome. For quality control, original cDNAs and RT-qPCR will be used in the validation. cDNAs will be amplified using TaqMan Universal PCR Master Mix. The expression levels will be normalized with TBP or GAPDH. Small interfering RNAs targeting candidate genes will be used. For metabolomics we will perform chromatography, mass spectrometry, immunohistochemistry, Western blot, and ELISA on specimens. Genetic calculations on Hardy–Weinberg equilibrium and linkage disequilibrium will be performed with Arlequin (version 3.5) using SPSS Statistics (version 20.0, IBM).

### Implementation of the study plan

The enrollment of the study subjects has started during 2021, and the clinical trial is ongoing. The end of the trial and preliminary results will be estimated during 2025. Further biochemical analyses will be performed later in the future.

## Discussion

In the era of advancing biological treatments for severe asthma and CRSwNP, significant progress has been made in the disease control of the patients also with the most difficult phenotypes, such as N-ERD. However, in clinical trials with biologicals, patients with N-ERD have often comprised less than 30% of the CRSwNP patient population (14, 18, 20, 21).

Several retrospective cohort studies have examined the effects of biologics on N-ERD patients. A retrospective study analyzed 43 NERD patients treated with either biologics or ATAD, all post-ESS (22). Of these, 45.7% received biologics (omalizumab 57%, dupilumab 28.5%, mepolizumab 9.5%, benralizumab 5%), while 54.3% underwent ATAD. After 1 year, biologic-treated patients showed an 82% reduction in Meltzer polyp scores, nearly 50% improvement in SNOT-22 scores, and a 42% reduction in FeNO within 6 months, alongside significantly better asthma control (ACT scores improved from 15.2 to 20.42). Biologics provided greater improvements in nasal and asthma outcomes than ATAD, with evidence suggesting a specific impact on lower airways (22).

Mullur et al. examined the use and experiences of biologics and ATAD in 98 NERD patients from the Brigham and Women's Hospital NERD registry. Of these, 52 (53.0%) had used one or more biologics (omalizumab, mepolizumab, reslizumab, benralizumab, or dupilumab), with many switching therapies (23). Additionally, 84 (85.7%) tried ATAD, with 78 (79.6%) continuing it. Twenty-four patients (24.4%) reported using both biologics and ATAD simultaneously. Patients using both therapies perceived ATAD as less effective compared to those using it alone (23).

No head-to head comparisons have been conducted either between different biologicals, but in a single center retrospective study of 74 N-ERD patients, dupilumab was the most beneficial clinically compared to omalizumab, mepolizumab and reslizumab (24). All patients who used biologics for at least 1 year in this cohort reduced their use of systemic corticosteroids with a median reduction of 178 mg (25). Oykhman et al. conducted a systematic review and network meta-analysis comparing biologicals and ASA desensitization for CRSwNP treatment and showed that dupilumab had the most beneficial effects for various outcomes examined (26).

The data for anti-IL-5(R) treatment and N-ERD are, however, mixed. In SYNAPSE phase 3 trial, N-ERD favored mepolizumab over placebo, but the symptom improvement was not significant (14). In OSTRO phase 3 trial, the nasal polyp score of N-ERD patients improved, but time to first ESS was not significantly different from placebo (23). To date, only small case series and retrospective cohorts of less than 15 patients that have studied mepolizumab and N-ERD (27), and no other double-blinded trials have been conducted to our knowledge.

The study protocol has some limitations that need discussion. Compared to previous RCTs, this study includes subjects with lower SNOT-22 score. This was intended to include patients with clinically significant symptoms while also capturing cases where patients may underestimate their sinonasal symptoms despite having severe disease. However, we acknowledge that this criterion may limit the comparability of our findings with previous studies. Therefore, *post hoc* analyses with a higher baseline mean SNOT-22 score may be necessary. The rationale for stool sample collection is to conduct a pilot study exploring potential links between the gut microbiome, disease severity, and treatment response in N-ERD. We acknowledge that factors such as antibiotic use may introduce confounders, and these will be accounted for in our data analysis. Due to logistical and funding constraints, our current follow-up period is limited. Yet we expect to observe significant changes even within the short follow-up period by utilizing statistical methods such as mixed effect models. We plan to develop a new study protocol to track patient outcomes using medical record data.

Taken together, based on literature of retrospective cohorts, mepolizumab among other biologics has demonstrated efficacy and safety in N-ERD patients and has shown superiority in both safety and efficacy over ATAD and oral corticosteroids. Although some *post hoc* analysis of clinical trials exists, there remains a lack of prospective data on the effects of mepolizumab on N-ERD.

Our randomized double-blind controlled clinical trial assesses the safety and efficacy of mepolizumab in the treatment of patients with N-ERD, CRSwNP and asthma. We will assess general and disease specific quality of life and disease control questionnaires, sense of smell, endoscopic nasal polyp score, lung function, absenteeism, and days of decreased productivity during work, as well as the use of treatment resources. Adverse events will be closely monitored throughout the study. By addressing these gaps, our study will bring valuable insights into the efficacy of mepolizumab for this severe subgroup of patients, that have often experienced treatment failure with other treatments.
